# Rice Bran Supplement Containing a Functional Substance, the Novel Peptide Leu-Arg-Ala, Has Anti-Hypertensive Effects: A Double-Blind, Randomized, Placebo-Controlled Study

**DOI:** 10.3390/nu11040726

**Published:** 2019-03-28

**Authors:** Yutaro Ogawa, Naohisa Shobako, Ikuo Fukuhara, Hisao Satoh, Etsuko Kobayashi, Takashi Kusakari, Makoto Suwa, Motonobu Matsumoto, Atsushi Ishikado

**Affiliations:** 1 Health Care R&D, Sunstar Inc., Takatsuki, Osaka 569-1195, Japan; yuutarou.ogawa@jp.sunstar.com (Y.O.); naohisa.shobako@jp.sunstar.com (N.S.); etsuko.kobayashi@jp.sunstar.com (E.K.); takashi.kusakari@jp.sunstar.com (T.K.); makoto.suwa@jp.sunstar.com (M.S.); motonobu.matsumoto@jp.sunstar.com (M.M.); 2 Fukuhara Clinic, Eniwa, Hokkaido 061-1351, Japan; i-feniwa@gray.plala.or.jp; 3 New Drug Research Center Inc., Eniwa, Hokkaido 061-1405, Japan; h-satoh@ndrcenter.co.jp

**Keywords:** double-blind randomized placebo-controlled study, anti-hypertensive effect, novel peptide, rice bran, high-normal blood pressure

## Abstract

The anti-hypertensive effect of processed rice bran (PRB) was recently reported, for which the novel peptide Leu-Arg-Ala (LRA) was identified as the functional substance. The purpose of this study was to assess the anti-hypertensive effects of a rice bran supplement containing PRB in individuals with high-normal blood pressure (systolic blood pressure (SBP): 130–139 mmHg and/or diastolic blood pressure (DBP): 85–89 mmHg) or grade 1 hypertension (SBP: 140–159 mmHg and/or DBP: 90–99 mmHg). One hundred individuals with high-normal blood pressure or grade 1 hypertension were recruited to participate in this double-blind, randomized, placebo-controlled study. Participants were randomly allocated to the placebo group (*n* = 50) or the test group (*n* = 50). Each group took four test tablets (43 μg LRA/day) or four placebo tablets daily. The decrease in blood pressure in the test group compared with the placebo group was the primary outcome. Adverse events were recorded and hematological/urinary parameters measured to determine the safety of the supplement, which was the secondary outcome. In total, 87 participants completed the study. The SBP of the test group at 12 weeks was significantly lower than that of the placebo group (*p* = 0.0497). No serious adverse events were observed. Daily consumption of a rice bran supplement containing PRB can safely improve mildly elevated blood pressure.

## 1. Introduction

Approximately one billion people worldwide suffer from hypertension, which is thought to be the main modifiable risk factor for cardiovascular disease [1,2]. Hypertension is also the most common lifestyle-related disease in Japan, where 34.6% of men and 24.8% of women have a systolic blood pressure (SBP) exceeding 140 mmHg [3]. Not only grade 1 hypertension (SBP: 140–159 mmHg/diastolic blood pressure (DBP): 90–99 mmHg), but also high-normal blood pressure (SBP: 130–139 mmHg/DBP: 85–89 mmHg), increase the risk of developing cardiovascular disease [4] (grade 1 hypertension and high-normal blood pressure are defined in the Japanese Society of Hypertension guidelines for the management of hypertension [5]). Interventions involving lifestyle modifications, such as dietary alterations, can prevent hypertension. For individuals with high-normal blood pressure or grade 1 hypertension, blood pressure control based on diet can be important for maintaining their quality of life. Some substances derived from food, such as peptides and flavonoids, have been reported to reduce blood pressure in individuals with high-normal blood pressure, grade 1 hypertension, or both [6,7,8]. Some of these substances are marketed as ‘foods for specified health use’ (FOSHU) and are used to reduce the risk of lifestyle-related disease in Japan.

Rice (*Oryza sativa*) is one of the three major food grains consumed worldwide, and is a staple food for about half of the world’s population [9]. Rice bran is a by-product of rice polishing, and while its high protein content and protein efficiency ratio are well known, it is not well utilized in the food industry, as it is primarily used for rice oil extraction or as feedstuff [10,11,12]. Some proteins derived from food materials are used to produce anti-hypertensive peptides. Ile-Pro-Pro (IPP), Val-Pro-Pro (VPP), and Met-Lys-Pro (MKP) are derived from casein, a protein found in milk, and their anti-hypertensive effects have been shown in clinical studies in humans [6,13]. Rice bran protein has been identified as a source of anti-hypertensive peptides, and the anti-hypertensive effect of processed rice bran (PRB) has been demonstrated in a previous study [14]. A novel anti-hypertensive peptide Leu-Arg-Ala (LRA), hydrolyzed from vicilin-like seed storage protein At2g28490, was identified as the functional substance of PRB. Oral administration of a low dose (0.25 mg/kg) of LRA has a potent anti-hypertensive effect in spontaneously hypertensive rats (SHRs) [14]. It was also reported that LRA has potent vasorelaxant activity (EC50 = 0.1 μM; the most potent value identified for a grain-derived peptide to date), and that this is the main mechanism accounting for its anti-hypertensive effect [15].

The purpose of the present study was to determine whether a rice bran supplement containing PRB can lower blood pressure in humans with high-normal blood pressure or grade 1 hypertension. The safety of the intervention was also determined as the secondary endpoint. To these ends, a randomized, double-blind, placebo-controlled clinical trial with an intake period of 12 weeks was conducted.

## 2. Materials and Methods

The present study was designed and carried out following the CONSORT 2010 statement guidelines, and a completed copy of the checklist is provided in Table S1.

### 2.1. Study Design

**Figure 1:**
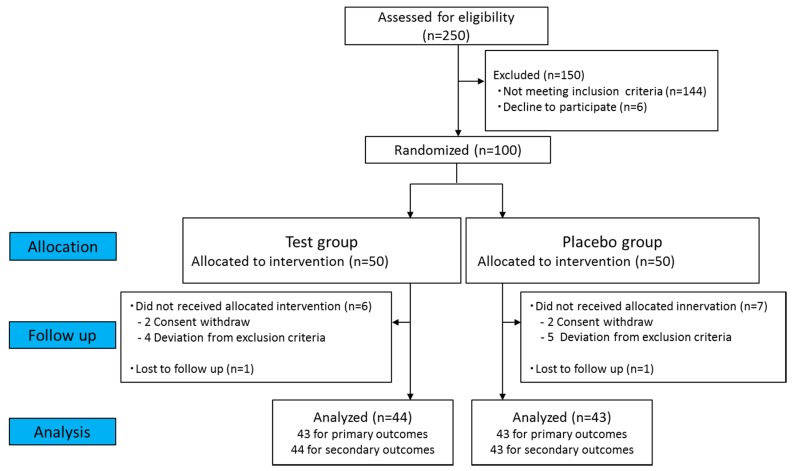
Flow chart of participant recruitment.

**Figure 2:**
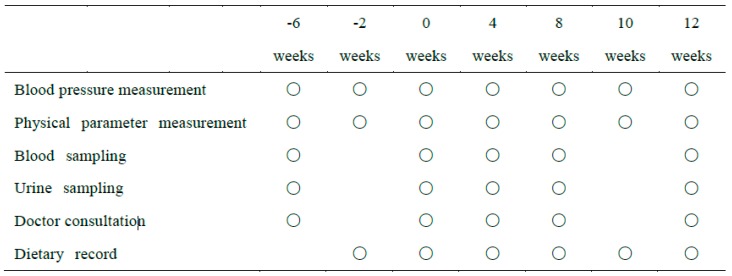
Test session schedule. Circles represent when data or samples were measured or collected.

The study was a 12-week, randomized, double-blind, placebo-controlled clinical trial (Figure 1 and Figure 2) carried out in accordance with the principles of the Declaration of Helsinki, with approval from the Institutional Review Board of Sunstar Inc. (17-FH-09), the Miyawaki Orthopedic Clinic Institutional Review Board (15000093), and the development subcontractors from the New Drug Research Center Inc. (Hokkaido, Japan), in accord with ethical guidelines for research on humans (Ministry of Education, Culture, Sports, Science and Technology; Ministry of Health, Labor and Welfare, Japan). The trial was registered in the UMIN Clinical Trials Registry (UMIN000028762).

### 2.2. Study Population

Hokkaido residents were recruited to participate in this study through a website from 8 June 2017 to 14 July 2017. Those who wished to participate in this trial were invited to Fukuhara Hospital (Hokkaido, Japan), where the details of the study and potential risks were thoroughly explained and written informed consent obtained. Inclusion criteria were age 45–64 years, high-normal blood pressure (SBP 130–139 mmHg and/or DBP 85–89 mmHg) or grade 1 hypertension (SBP 140–159 mmHg and/or DBP 90–99 mmHg) during the pre-observation period (at both 6 and 2 weeks prior to administration of the test supplement or placebo), and a body mass index (BMI) of less than 30.0 kg/m^2^. The following subjects were excluded: women with childbearing potential; pregnant or lactating women; individuals who had participated in other clinical trials within the 4 weeks preceding this study or who planned on participating in another study within 4 weeks of the end of this study; those with heart, liver, kidney, or thyroid disease (or complications thereof); those with a history of cardiovascular disease; those with diabetes; those with an allergy to brown rice, rice bran, or rice; those who had experienced adverse reactions to having blood drawn in the past; those who were excessive drinkers (average 30 mL equivalent/day alcohol) or heavy smokers (average 21 cigarettes/day or more); those on an extremely irregular diet (due to shift work or for other reasons); those on medications that may have affected this trial, such as anti-hypertensive drugs; those who regularly consumed specific supplements or foods with functional claims that may have affected this trial and who did not consent to refrain from consuming them for 1 week before preliminary test 2; those with secondary hypertension; those with a history of major gastrointestinal surgery such as stomach resection, gastrointestinal anastomosis, gastrointestinal surgery, or intestinal resection (excluding appendix resection); and those judged inappropriate for participation in the study based on the participant’s background, physical examination, and interview by the principal investigator. These exclusion criteria are based on previous clinical studies and the criteria of the NIPPON DATA 80 study [6,16,17,18,19,20].

### 2.3. Randomization

Participants were randomly assigned to two groups by the block randomization method, stratified by age and sex and subsequently stratified by condition (SBP or DBP). The randomization was performed by an allocation coordinator independent from the investigators, and the randomization code was kept in a sealed envelope to maintain blinding. Thus the investigators, participants, and other study personnel were blinded to the treatment assignments for the duration of the study.

### 2.4. Interventions

The trial was conducted in Hokkaido, Japan between 26 July and 9 December 2017. All study products (the test food and the placebo) were prepared as tablets that were identical in terms of appearance and flavor. The nutrient composition of the test products is described in Table 1. The rice bran supplements were prepared by rice bran hydrolyzation [14]. Rice bran was supplied by SUNBRAN (Yamagata, Japan). The LRA content in the test food was analyzed by liquid chromatography/mass spectrometry, and LRA was not detected in the placebo tablets. Participants in both groups took four tablets once a day with water after breakfast. Participants were asked to record the following information in a daily diary: drugs and FOSHUs that were consumed, schedules (such as when they awoke, slept, ate meals, and took the tablets), amount of alcohol consumed, and any symptoms that they noticed. Participants were advised to maintain their usual lifestyle habits, such as physical activity, throughout the study.

### 2.5. Outcomes

The primary outcomes of the study were SBP and DBP each week from the date on which the first dose of tablets was consumed. Safety, a secondary outcome, was evaluated by the number of adverse events, adverse event occurrence rate, number of side effects, and side effect occurrence rate.

### 2.6. Procedures

Blood pressure, pulse rate, and body temperature—Blood pressure, pulse rate, and body temperature were measured seven times in total: at 6 and 2 weeks before starting to take the tablets, on the day the participants started taking the tablets, and at 4, 8, 10, and 12 weeks after starting to take the tablets. Blood pressure was measured using an automatic sphygmomanometer (HEM-759P; Omron, Kyoto, Japan) based on the method described in the Japanese Society of Hypertension guidelines for the management of hypertension [5], when the subject had been seated at rest (feet uncrossed, no talking) for at least 10 min after arriving at the hospital. The average value of two measurements with similar values (difference < 5 mmHg) was recorded as the blood pressure. Before visiting for blood pressure measurements, participants were asked to do the following: eat dinner by 21:00 on the day before the test, refrain from alcohol and avoid eating and drinking after 21:00 on the day before the test (participants were allowed to drink approximately ~200 mL of water up to 2 h before the measurement was taken), and refrain from smoking until after the measurement was taken to avoid influence on the outcome values such as blood pressure, blood glucose, and blood lipids.

Weight and height—Body weight was measured a total of six times: at 6 weeks before starting to take the tablets, on the day that participants began taking the tablets, and at 4, 8, 10, and 12 weeks after starting to take the tablets. Body height was measured once—at 6 weeks before starting to take the tablets—and the measured value was used to calculate the BMI.

Laboratory tests—Laboratory tests were carried out five times in total: at 6 weeks before starting to take the tablets, on the day the participants started to take the tablets, and at 4, 8, and 12 weeks after starting to take the tablets. Blood (10 mL) and urine (7 mL) were collected.

For blood tests, white blood cell count, erythrocyte count, hemoglobin, hematocrit, platelet count, total protein, albumin, total bilirubin, aspartate aminotransferase, alanine aminotransferase, lactate dehydrogenase, alkaline phosphatase isozymes, γ-glutamyltransferase, creatine kinase isoenzyme, total cholesterol, high-density lipoprotein cholesterol, low-density lipoprotein cholesterol, triglycerides, fasting blood glucose, hemoglobin A1c (national glycohemoglobin standardization was only performed for preliminary test 1), uric acid, urea nitrogen, creatinine, sodium, chloride, potassium, and calcium were measured. Urinalysis was performed to determine specific gravity, pH, ketone bodies, occult blood reaction, urobilinogen (qualitative), bilirubin (qualitative), sugar (qualitative), protein (qualitative), sodium, creatinine, potassium, estimated daily urinary sodium excretion, and estimated daily urinary potassium excretion. Gram creatinine correction was performed for the estimated daily urinary sodium excretion and estimated daily urinary potassium excretion. These parameters were measured by an auto-analyzer (BM8040—JEOL, Tokyo, Japan; XN9100—Sysmex, Hyogo, Japan; or US3500—Eiken Chemical, Tokyo, Japan).

### 2.7. Statistical Analysis

Sample size was calculated based on the results from a previous study (a test performed to determine the optimum concentration) in which administration of 43 μg of LRA derived from 1 g of PRB significantly reduced blood pressure compared with the placebo group (unpublished data).

The parameters analyzed in this study are presented as means and standard deviation (SD). The efficacy analysis was based on the per-protocol set. The safety analysis was based on a modified intention-to-treat principle (full analysis set).

To compare the numerical data for the placebo group and the test group at each time point, an F-test of equality of variances was performed. In the case of equal variance Student’s *t*-test was performed, while in the case of unequal variance an Aspin–Welch *t*-test was performed. For the semi-quantitative values derived from the urinalysis, a Mann–Whitney test was performed. For all two-sided tests, the significance level was set at 5% or 1%.

For the efficacy analysis, a repeated-measures analysis of variance was also performed to compare the blood pressure at week 0 with the blood pressure at weeks 4, 8, 10, and 12 in each group. If a significant difference was found, the average values were compared by Dunnett’s method.

For the safety assessment, Fisher’s exact test was performed to detect differences between groups in terms of the number of side effects and number of adverse events. For the laboratory test data and physical findings, a repeated-measures analysis of variance was performed. If a significant difference was found, the average values were compared by Dunnett’s method. A Wilcoxon signed-rank test was performed for the semi-quantitative values obtained from the urinalysis.

A subgroup analysis was planned by dividing the subjects into two groups (high-normal blood pressure and grade 1 hypertension) according to the blood pressure values recorded when the participants started taking the tablets.

## 3. Results

### 3.1. Participants

In total, 250 participants were recruited and screened. One hundred participants were enrolled and randomly allocated to the test or placebo group (Figure 1). Four participants (two in the test group and two in the placebo group) withdrew before further assessment for personal reasons unrelated to the trial. Nine participants (four in the test group and five in the placebo group) did not fulfill the inclusion criteria, as their baseline blood pressures were not classified to grade 1 hypertension or high-normal hypertension. Two participants withdrew for reasons unrelated to the trial. Data from participants whose nutritional surveys and medication were judged by the principal investigator as having the potential to interfere with interpretation of the results were excluded before code breaking. The overall dropout rate was 13% (13 out of 100). The demographic characteristics and baseline data for each group are shown in Table 2. None of the participants were taking anti-hypertensive medication during the trial. Sixteen participants took agents such as intestinal drugs during the test period. As the principal investigator judged that none of these were related to the test food, these participants were included in the statistical analysis. The estimated salt intake of each group was not significantly different between both groups during the test period (Table S2).

### 3.2. Blood Pressure Response

**Figure 3:**
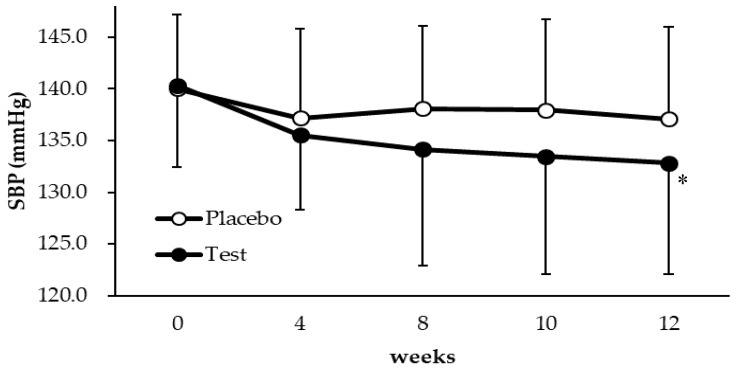
Changes in SBP over 12 weeks in individuals who received the test food or the placebo. * Significantly different from the placebo group (*p* < 0.05). Each value is expressed as the mean ± SD.

The mean SBP values at each time point in all subjects with high-normal blood pressure and grade 1 hypertension in the test (*n* = 43) and placebo (*n* = 43) groups are shown in Table 3 and Figure 3. The mean SBP in the test group was significantly lower than that in the placebo group at 12 weeks (*p* = 0.0497). There was no difference in effectiveness between male and female participants. In contrast, the mean DBP did not differ significantly from that of the placebo group at any subsequent time point.

**Figure 4:**
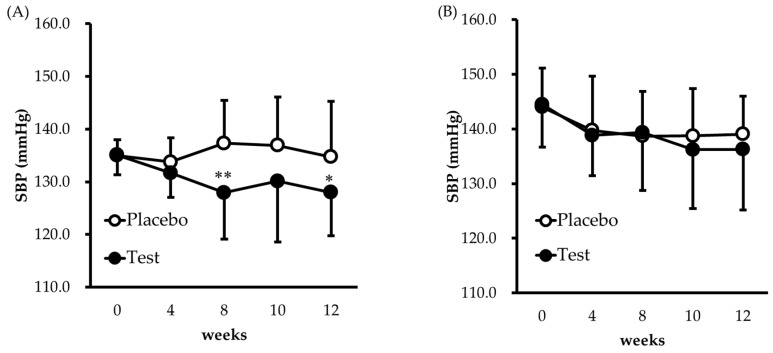
Changes in SBP over 12 weeks in participants who received the test food or the placebo (**A**) high-normal BP; (**B**) grade 1 hypertension. * *p* < 0.05, ** *p* < 0.01, significantly different from the placebo group. Each value is expressed as the mean ± SD.

Next, stratified analysis was conducted. The results for the participants with high-normal blood pressure are shown in Figure 4A and Table 4A. Significant reductions in SBP were observed in the high-normal blood pressure subgroup at week 8 (*p* = 0.0014) and week 12 (*p* = 0.0397) compared with the placebo group, while the DBP did not differ significantly from that of the placebo group. In the grade 1 hypertension subgroup, no significant difference was observed (Figure 4B and Table 4B).

### 3.3. Safety

A total of 17 adverse events were reported throughout the study, with 12 reported by the placebo group and 5 by the test group. All reported adverse events are listed in Table S3. The principal investigator judged that none of these adverse events were related to the test food. No significant differences in body weight and risk factors (BMI, blood lipid, uric acid, blood glucose, and liver function) between the placebo group and the test group were observed at the end of the trial (Table 5). The values of all of the parameters measured at 0 and 12 weeks to assess the safety of the intervention are shown in Table S4.

## 4. Discussion

In the present study, it was demonstrated that taking an anti-hypertensive food product for 12 weeks reduced SBP. Hypertension is one of the important risk factors for cardiovascular disease. It is well established that medical therapies for hypertension can reduce the rate of death from cardiovascular disease, myocardial infarction, and stroke [21,22]. Thus, lowering blood pressure through the use of a food-derived material could represent a powerful approach for preventing cardiovascular disease and maintaining quality of life. Food-derived substances such as polyphenols and peptides have been shown to have anti-hypertensive effects in human clinical studies [23,24]. For example, digestion of milk casein containing the hypotensive peptide Met-Lys-Pro (MKP) showed anti-hypertensive activity in humans with high-normal blood pressure [6]. In this study, hydrolyzed rice bran protein containing a novel peptide Leu-Arg-Ala (LRA) as a functional substance was evaluated. LRA reduced SBP in SHRs and its minimal effective dose was 0.25 mg/kg, which is comparable with the pharmaceutical dose [14]. As shown in Figure 3 and Table 3, the test food caused a significant reduction in SBP compared with the placebo at 12 weeks. Administration of the test food also resulted in a significant reduction in SBP (−7.5 mmHg, *p* < 0.05) compared with baseline. Twelve-week interventions with treatments such as angiotensin II type 1 receptor blockers and DASH (Dietary Approaches to Stop Hypertension) diets reduced SBP by 5–10 mmHg in patients with grade 1 hypertension [25,26]. The decrease in SBP induced by the test food is potentially comparable with that induced by these treatments. This clinical study supports our hypothesis that the rice bran supplements containing LRA are effective anti-hypertensive agents in humans.

Previous clinical studies have shown that rice bran reduces SBP when combined with other interventions such as energy restriction or the administration of sesame oil [27,28]. This is the first study to show that a rice bran-based food material alone can reduce blood pressure without being combined with another intervention.

The activity of most anti-hypertensive food-derived peptides is based on angiotensin I-converting enzyme (ACE) inhibition [29,30,31]. Some peptides have been shown to exert their anti-hypertensive effects through nitric oxide (NO)-mediated vasorelaxation. NO is an important vasodilator that is produced from arginine in the endothelial layer and causes relaxation of vascular smooth muscle. LRA induces vasorelaxation by activating endothelial NO synthase. LRA also induces vasorelaxation of the mesenteric artery in SHRs in a dose-dependent manner, and its EC_50_ value was reported to be 0.1 μM, the most potent value for all known grain-derived anti-hypertensive materials [15]. Chlorogenic acid and peptides derived from chicken collagen hydrate are also reported to improve NO-mediated vasorelaxation and significantly reduce SBP, but not DBP [32,33]. Physical activities such as aerobic exercise and isometric exercise also promote NO bioavailability in the endothelial layer and are likely to reduce SBP more than DBP [34]. Taken together, these studies suggest that NO-mediated vasorelaxation lowers SBP more effectively than DBP, which also appears to be the case for the anti-hypertensive effect of rice bran supplement containing the functional substance LRA tested in this study. Enhanced NO-mediated vasorelaxation improves vascular function, as measured by flow-mediated dilation. Further studies are needed to investigate whether our food-derived product also improves vascular function.

In this study, a difference in effectiveness between male and female participants was not observed, whereas some studies have shown estrogen to activate the NO system and play a key role in such a difference [35]. LRA is also reported to show a less potent but specific ACE-inhibitory activity, and other anti-hypertensive pathways might also contribute to the hypotensive activity [14]. Therefore, the anti-hypertensive effect of LRA might not be affected by sex difference.

Our stratified analysis showed that test food reduced SBP in subjects with high-normal blood pressure (Figure 4A). Several studies have shown that high-normal blood pressure is a risk factor for cardiovascular disease. For example, the Framingham study found that there is an elevated risk of cardiovascular complications in patients with an SBP of ≥120 mmHg [36], while the NIPPON DATA 80 study reported that the rate of stroke and circulatory disease is higher in subjects with high-normal blood pressure compared with those with normal blood pressure [37]. In the SPRINT study, significant reductions in cardiovascular events and all-cause mortality were observed in an intensive therapy group with an SBP of <120 mmHg in comparison with a standard therapy group with an SBP of <140 mmHg [38]. As these studies demonstrate, it is important to prevent patients with high-normal blood pressure from progressing to serious disease, and especially important to reduce the SBP in these patients. In the present study, SBP was significantly lowered by the test food even in subjects with high-normal blood pressure, suggesting that the test material may help prevent the development of cardiovascular disorders. Otherwise a significant difference was not observed in the grade 1 hypertension subgroup. As shown in Figure 4B, blood pressure was not stable in this subgroup and the effectiveness was not accurately evaluated. In addition, it has been reported that the anti-hypertensive effect of a milk casein diet including MKP was shown not in a grade 1 hypertension subgroup but in a high-normal blood pressure subgroup [6]. This result suggests that food-derived anti-hypertensive peptide might be more effective for persons with high-normal blood pressure, although further study is needed to determine the anti-hypertensive effect of PRB, including LRA for patients with grade 1 hypertension.

As shown in Table 5 and Supplementary Tables S3 and S4, the prevalence of adverse events was low and the rice bran supplement did not affect body weight, blood lipid level, and blood glucose metabolism. Safety was also demonstrated previously by an excessive consumption test (5 g PRB/day, 5-fold intake over this study) (unpublished data). These results suggest that the rice bran supplement could be utilized for functional foods such as FOSHU and safely prevent hypertension. In a previous study, the anti-hypertensive effect of milk casein hydrate for normotensive humans was determined, whereby no hypotensive effect was observed [39]. In similar manner, the rice bran supplement also might not lead to excessive blood pressure reduction in normotensive individuals. Further studies are required to elucidate the effect of anti-hypertensive food materials in normotensive humans.

The strengths of this study are three-fold. First, this is the first report showing that a rice bran supplement alone can reduce blood pressure without being combined with another intervention. The dietary intervention in this study appears to be feasible for clinical practice, especially in Asian countries where rice is a staple food. It was also shown that the anti-hypertensive effect of the rice bran supplement was independent of any reduction in body weight, decrease in blood lipid level, or change in glucose metabolism. Second, the SBP was effectively reduced in human subjects with high-normal blood pressure and grade 1 hypertension in a trial with a relatively small but sufficient sample size. Third, the safety of the rice bran supplement was demonstrated, as it was associated with a low prevalence of adverse events. However, this study also has some limitations. The intervention period lasted only 12 weeks, and only one dose of the rice bran supplement was tested. In addition, the mechanism underlying the anti-hypertensive effect observed was not investigated. It is also possible that the test food contained one or more functional substances other than LRA. Finally, only individuals with high-normal blood pressure and grade 1 hypertension, without other serious disease such as diabetes, were assessed in this trial; the effectiveness of this intervention should also be tested in patients with grade 2 and 3 hypertension, other diseases, or individuals with a smoking habit.

To sum up, a 12-week intervention involving LRA derived from PRB effectively reduced the SBP in individuals with grade 1 hypertension and high-normal blood pressure. Stratified subgroup analysis showed that this anti-hypertensive effect was statistically significant in the subgroup with high-normal blood pressure.

## 5. Conclusions

In conclusion, it has been shown for the first time that 12-week administration of a rice bran supplement containing the functional peptide LRA significantly reduced the SBP in patients with grade 1 hypertension and high-normal blood pressure in comparison with a placebo group in a double-blind, randomized, placebo-controlled study. Stratified analysis showed that the rice bran supplement had a potent anti-hypertensive effect on subjects with high-normal blood pressure, indicating that the rice bran supplement could be useful for preventing the progression of patients with pre-hypertension to grade 1 hypertension.

## Figures and Tables

**Table 1:** Composition of the tablets used in this trial.

	Test	Placebo
Calories (kcal/day)	4.45	5.42
Protein (g/day)	0.26	0.00
Fat (g/day)	0.05	0.04
Carbohydrate (g/day)	0.75	1.27
Sodium (mg/day)	7.39	0.00
LRA (μg/mL)	43.00	0.00

**Table 2:** Demographic characteristics and baseline data of the participants.

	Placebo		Test		*p*
Male/Female	16/27		15/29		
Age (years)	54.1	(6.0)	53.8	(5.7)	0.7697
Body weight (kg)	65.1	(9.8)	63.0	(11.3)	0.3641
Body mass index (kg/m^2^)	24.8	(2.7)	24.3	(3.0)	0.4534
SBP (mmHg)	141.9	(8.5)	141.0	(8.5)	0.6242
DBP (mmHg)	88.9	(6.4)	89.4	(7.0)	0.7150
Body temperature (°C)	36.5	(0.3)	36.4	(0.3)	0.2501
Pulse rate (beats/min)	69.9	(9.6)	71.0	(9.8)	0.6157
Total cholesterol (mg/dL)	217.3	(32.1)	213.6	(29.4)	0.5722
HDL-C (mg/dL)	65.0	(13.7)	64.9	(16.8)	0.9725
LDL-C (mg/dL)	130.3	(25.8)	126.1	(29.2)	0.4804
Triglycerides (mg/dL)	110.3	(53.4)	113.3	(68.8)	0.8216
Uric acid (mg/dL)	5.4	(1.2)	5.2	(1.2)	0.5373
Fasting blood glucose (mg/dL)	90.8	(8.6)	89.8	(7.2)	0.5697
ALT (GPT) (U/L)	21.1	(9.8)	25.6	(19.1)	0.1742

Each value is expressed as the mean (SD). SBP: systolic blood pressure; DBP: diastolic blood pressure; HDL-C: high-density lipoprotein cholesterol; LDL-C: low-density lipoprotein cholesterol; ALT: alanine aminotransferase; GPT: glutamic-pyruvic transaminase.

**Table 3:** Changes in SBP and DBP in all subjects with high-normal blood pressure and grade 1 hypertension.

		Placebo				Test				
	Weeks	*n*	Mean	(SD)		*n*	Mean	(SD)	ΔBP	*p*
SBP	0	43	140.0	(7.2)		43	140.3	(7.9)	0.3 (−2.9 to 3.5)	0.8528
	12	42	137.1	(8.9)		41	132.8	(10.7)	−4.3 (−8.6 to −0.006)	0.0497
DBP	0	43	89.4	(6.1)		43	90.3	(5.6)	0.9 (−1.7 to 3.4)	0.4985
	12	42	87.0	(5.8)		41	87.0	(8.0)	−0.1 (−3.1 to 3.0)	0.9687

Each value is expressed as the mean (SD). ΔBP indicates the difference in the averages of the test group and the placebo group (95% confidence interval). SBP: systolic blood pressure; DBP: diastolic blood pressure.

**Table 4:** Results from the stratified analysis.

**(A) High-normal BP**		**Placebo**				**Test**				
	**Week**	***n***	**Mean**	**(SD)**		***n***	**Mean**	**(SD)**	**Δ BP**	***p***
SBP	0	19	135.0	(3.0)		19	135.1	(3.7)	0.1 (−2.1 to 2.3)	0.9426
	12	19	134.7	(10.5)		17	128.0	(8.2)	−6.8 (−13.2 to −0.3)	0.0397
DBP	0	19	84.3	(4.2)		19	86.0	(4.0)	1.7 (−1.0 to 4.4)	0.2205
	12	19	83.6	(5.3)		17	82.0	(7.6)	−1.6 (−6.0 to 2.8)	0.4631
**(B) Grade 1 HT**		**Placebo**				**Test**				
	**Week**	***n***	**Mean**	**(SD)**		***n***	**Mean**	**(SD)**	**ΔBP**	***p***
SBP	0	24	144.0	(7.1)		24	144.5	(7.8)	0.5 (−3.9 to 4.8)	0.8247
	12	23	139.1	(6.9)		24	136.3	(11.1)	−2.8 (−8.3 to 2.6)	0.2995
DBP	0	24	93.4	(4.2)		24	93.7	(4.2)	0.2 (−2.2 to 2.7)	0.8495
	12	23	89.8	(4.7)		24	90.5	(6.3)	0.6 (−2.6 to 3.9)	0.6989

Each value is expressed as the mean (SD). High-normal BP: high-normal blood pressure; Grade 1 HT: grade 1 hypertension. ΔBP indicates the difference in the averages of the test group and the placebo group (95% confidence interval).

**Table 5:** Body weight, risk factors, and representative values of liver function at 12 weeks.

	Placebo		Test		*p*
Body weight (kg)	65.3	(9.7)	64.1	(11.8)	0.5882
Body mass index (kg/m^2^)	24.9	(2.8)	24.7	(3.1)	0.7985
Total cholesterol (mg/dL)	211.4	(33.2)	215.5	(31.8)	0.5642
HDL-C (mg/dL)	65.8	(14.5)	65.3	(18.5)	0.9091
LDL-C (mg/dL)	126.1	(25.3)	128.6	(31.9)	0.6868
Triglycerides (mg/dL)	98.0	(58.5)	107.7	(72.0)	0.4969
Uric acid (mg/dL)	5.4	(1.1)	5.2	(1.4)	0.3613
Fasting blood glucose (mg/dL)	90.7	(8.2)	92.2	(9.3)	0.4495
ALT (GPT) (U/L)	23.1	(9.9)	29.0	(29.7)	0.2184

Each value is expressed as the mean (SD). HDL-C: high-density lipoprotein cholesterol; LDL-C: low-density lipoprotein cholesterol; ALT: alanine aminotransferase; GPT: glutamic-pyruvic transaminase.

## Supplementary Materials

The following are available online at https://www.mdpi.com/2072-6643/11/4/726/s1, Table S1: CONSORT 2010 checklist of information to include when reporting a randomized trial, Table S2: Measured urine sodium and creatinine, estimated salt intake of participants, Table S3: All of adverse events reported throughout the study. Table S4: Values of body weight, BMI, hematological parameters measured in this study.
