# Supplementary material for: Rice Bran Supplement Containing a Functional Substance, the Novel Peptide Leu-Arg-Ala, Has Anti-Hypertensive Effects: A Double-Blind, Randomized, Placebo-Controlled Study
Source: Nutrients. 2019 Mar 28;11(4):726. doi: 10.3390/nu11040726 (PMC6521331; doi:10.3390/nu11040726)
Supplement: Supplementary file 1 [file nutrients-11-00726-s001.zip › TableS2_DailySodiumIntake .pptx]

## Slide 1
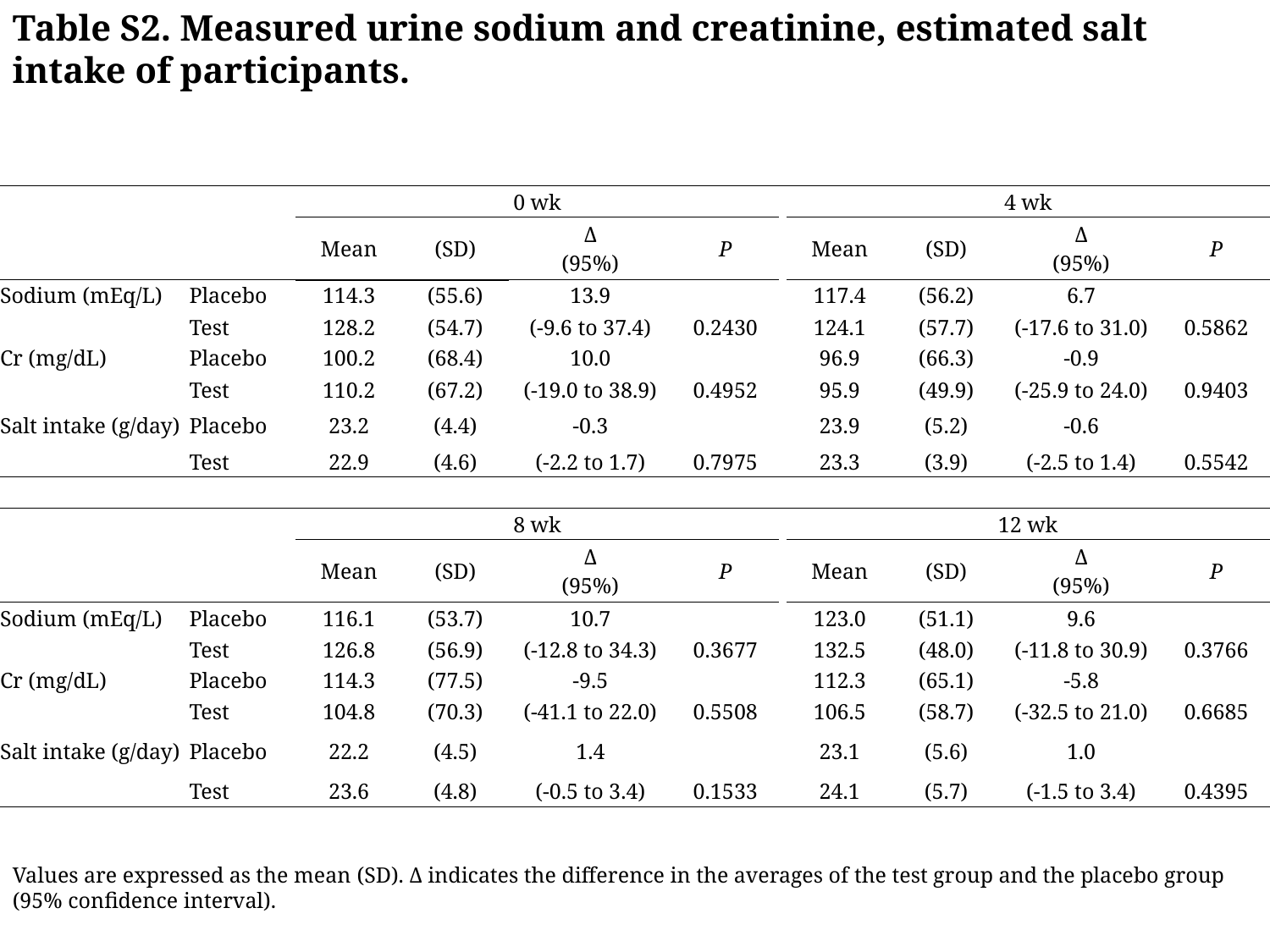

Table S2. Measured urine sodium and creatinine, estimated salt intake of participants.
| | | 0 wk | | | | | 4 wk | | | |
| --- | --- | --- | --- | --- | --- | --- | --- | --- | --- | --- |
| | | Mean | (SD) | Δ (95%) | P | | Mean | (SD) | Δ (95%) | P |
| Sodium (mEq/L) | Placebo | 114.3 | (55.6) | 13.9 | | | 117.4 | (56.2) | 6.7 | |
| | Test | 128.2 | (54.7) | (-9.6 to 37.4) | 0.2430 | | 124.1 | (57.7) | (-17.6 to 31.0) | 0.5862 |
| Cr (mg/dL) | Placebo | 100.2 | (68.4) | 10.0 | | | 96.9 | (66.3) | -0.9 | |
| | Test | 110.2 | (67.2) | (-19.0 to 38.9) | 0.4952 | | 95.9 | (49.9) | (-25.9 to 24.0) | 0.9403 |
| Salt intake (g/day) | Placebo | 23.2 | (4.4) | -0.3 | | | 23.9 | (5.2) | -0.6 | |
| | Test | 22.9 | (4.6) | (-2.2 to 1.7) | 0.7975 | | 23.3 | (3.9) | (-2.5 to 1.4) | 0.5542 |
| | | | | | | | | | | |
| | | 8 wk | | | | | 12 wk | | | |
| | | Mean | (SD) | Δ (95%) | P | | Mean | (SD) | Δ (95%) | P |
| Sodium (mEq/L) | Placebo | 116.1 | (53.7) | 10.7 | | | 123.0 | (51.1) | 9.6 | |
| | Test | 126.8 | (56.9) | (-12.8 to 34.3) | 0.3677 | | 132.5 | (48.0) | (-11.8 to 30.9) | 0.3766 |
| Cr (mg/dL) | Placebo | 114.3 | (77.5) | -9.5 | | | 112.3 | (65.1) | -5.8 | |
| | Test | 104.8 | (70.3) | (-41.1 to 22.0) | 0.5508 | | 106.5 | (58.7) | (-32.5 to 21.0) | 0.6685 |
| Salt intake (g/day) | Placebo | 22.2 | (4.5) | 1.4 | | | 23.1 | (5.6) | 1.0 | |
| | Test | 23.6 | (4.8) | (-0.5 to 3.4) | 0.1533 | | 24.1 | (5.7) | (-1.5 to 3.4) | 0.4395 |
Values are expressed as the mean (SD). Δ indicates the difference in the averages of the test group and the placebo group (95% confidence interval).
