# Supplementary material for: Rice Bran Supplement Containing a Functional Substance, the Novel Peptide Leu-Arg-Ala, Has Anti-Hypertensive Effects: A Double-Blind, Randomized, Placebo-Controlled Study
Source: Nutrients. 2019 Mar 28;11(4):726. doi: 10.3390/nu11040726 (PMC6521331; doi:10.3390/nu11040726)
Supplement: Supplementary file 1 [file nutrients-11-00726-s001.zip › TableS3_Advere_events.pptx]

## Slide 1
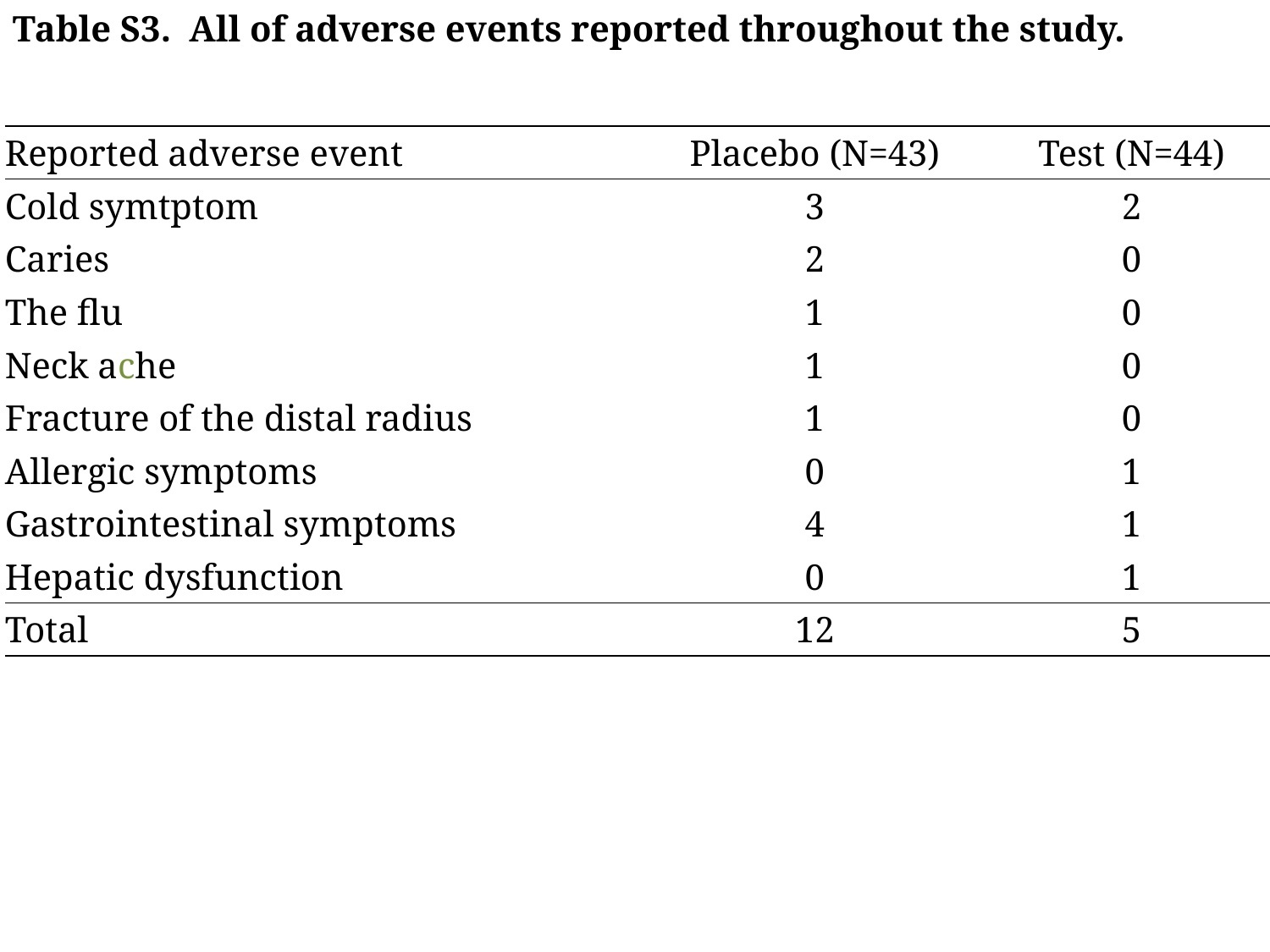

Table S3. All of adverse events reported throughout the study.
| Reported adverse event | Placebo (N=43) | Test (N=44) |
| --- | --- | --- |
| Cold symtptom | 3 | 2 |
| Caries | 2 | 0 |
| The flu | 1 | 0 |
| Neck ache | 1 | 0 |
| Fracture of the distal radius | 1 | 0 |
| Allergic symptoms | 0 | 1 |
| Gastrointestinal symptoms | 4 | 1 |
| Hepatic dysfunction | 0 | 1 |
| Total | 12 | 5 |
