# Supplementary material for: Rice Bran Supplement Containing a Functional Substance, the Novel Peptide Leu-Arg-Ala, Has Anti-Hypertensive Effects: A Double-Blind, Randomized, Placebo-Controlled Study
Source: Nutrients. 2019 Mar 28;11(4):726. doi: 10.3390/nu11040726 (PMC6521331; doi:10.3390/nu11040726)
Supplement: Supplementary file 1 [file nutrients-11-00726-s001.zip › TableS4_Result_table.pptx]

## Slide 1
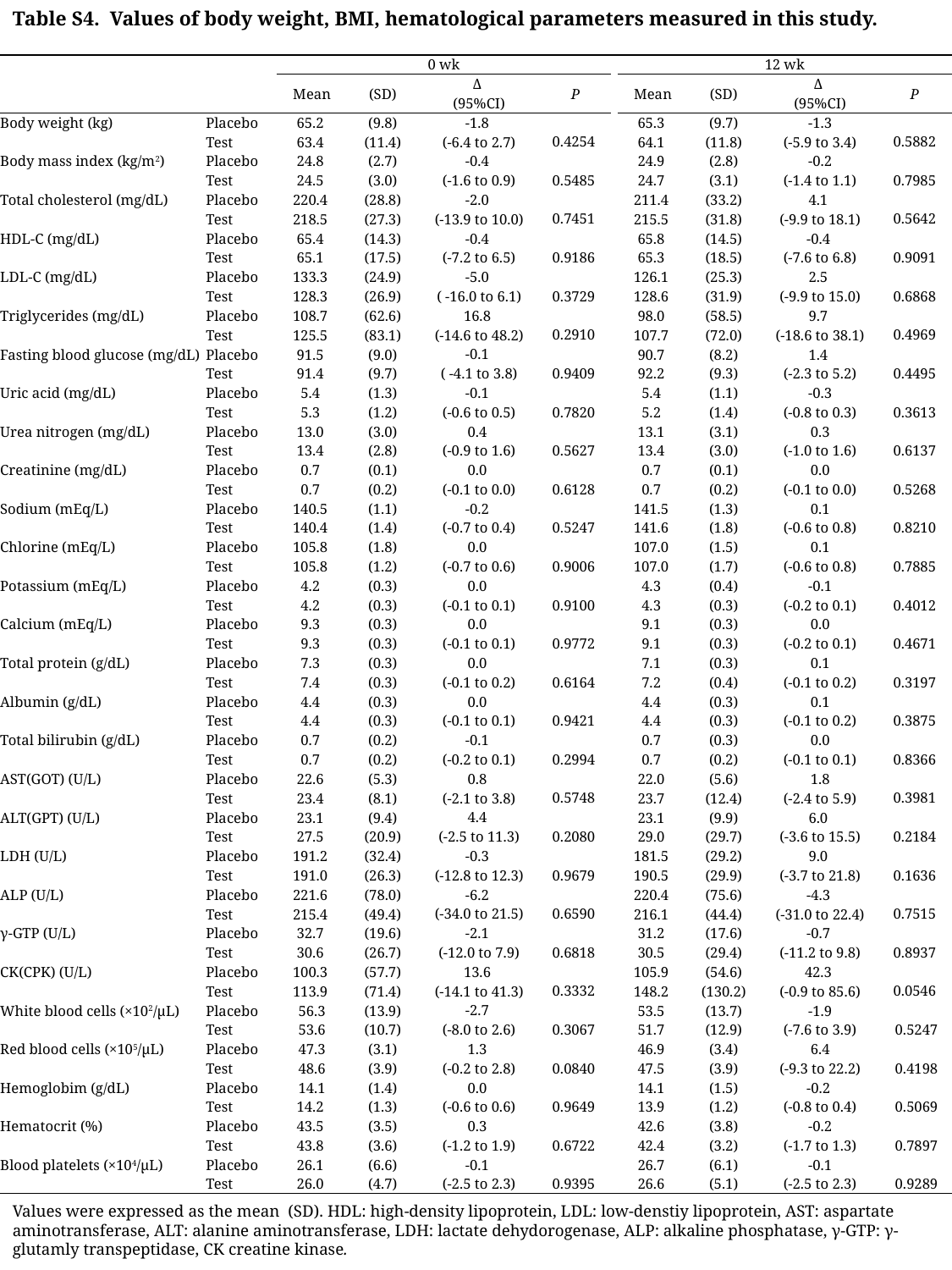

Table S4. Values of body weight, BMI, hematological parameters measured in this study.
| | | 0 wk | | | | | 12 wk | | | |
| --- | --- | --- | --- | --- | --- | --- | --- | --- | --- | --- |
| | | Mean | (SD) | Δ (95%CI) | P | | Mean | (SD) | Δ (95%CI) | P |
| Body weight (kg) | Placebo | 65.2 | (9.8) | -1.8 | 0.4254 | | 65.3 | (9.7) | -1.3 | 0.5882 |
| | Test | 63.4 | (11.4) | (-6.4 to 2.7) | | | 64.1 | (11.8) | (-5.9 to 3.4) | |
| Body mass index (kg/m2) | Placebo | 24.8 | (2.7) | -0.4 | 0.5485 | | 24.9 | (2.8) | -0.2 | 0.7985 |
| | Test | 24.5 | (3.0) | (-1.6 to 0.9) | | | 24.7 | (3.1) | (-1.4 to 1.1) | |
| Total cholesterol (mg/dL) | Placebo | 220.4 | (28.8) | -2.0 | 0.7451 | | 211.4 | (33.2) | 4.1 | 0.5642 |
| | Test | 218.5 | (27.3) | (-13.9 to 10.0) | | | 215.5 | (31.8) | (-9.9 to 18.1) | |
| HDL-C (mg/dL) | Placebo | 65.4 | (14.3) | -0.4 | 0.9186 | | 65.8 | (14.5) | -0.4 | 0.9091 |
| | Test | 65.1 | (17.5) | (-7.2 to 6.5) | | | 65.3 | (18.5) | (-7.6 to 6.8) | |
| LDL-C (mg/dL) | Placebo | 133.3 | (24.9) | -5.0 | 0.3729 | | 126.1 | (25.3) | 2.5 | 0.6868 |
| | Test | 128.3 | (26.9) | ( -16.0 to 6.1) | | | 128.6 | (31.9) | (-9.9 to 15.0) | |
| Triglycerides (mg/dL) | Placebo | 108.7 | (62.6) | 16.8 | 0.2910 | | 98.0 | (58.5) | 9.7 | 0.4969 |
| | Test | 125.5 | (83.1) | (-14.6 to 48.2) | | | 107.7 | (72.0) | (-18.6 to 38.1) | |
| Fasting blood glucose (mg/dL) | Placebo | 91.5 | (9.0) | -0.1 | 0.9409 | | 90.7 | (8.2) | 1.4 | 0.4495 |
| | Test | 91.4 | (9.7) | ( -4.1 to 3.8) | | | 92.2 | (9.3) | (-2.3 to 5.2) | |
| Uric acid (mg/dL) | Placebo | 5.4 | (1.3) | -0.1 | 0.7820 | | 5.4 | (1.1) | -0.3 | 0.3613 |
| | Test | 5.3 | (1.2) | (-0.6 to 0.5) | | | 5.2 | (1.4) | (-0.8 to 0.3) | |
| Urea nitrogen (mg/dL) | Placebo | 13.0 | (3.0) | 0.4 | 0.5627 | | 13.1 | (3.1) | 0.3 | 0.6137 |
| | Test | 13.4 | (2.8) | (-0.9 to 1.6) | | | 13.4 | (3.0) | (-1.0 to 1.6) | |
| Creatinine (mg/dL) | Placebo | 0.7 | (0.1) | 0.0 | 0.6128 | | 0.7 | (0.1) | 0.0 | 0.5268 |
| | Test | 0.7 | (0.2) | (-0.1 to 0.0) | | | 0.7 | (0.2) | (-0.1 to 0.0) | |
| Sodium (mEq/L) | Placebo | 140.5 | (1.1) | -0.2 | 0.5247 | | 141.5 | (1.3) | 0.1 | 0.8210 |
| | Test | 140.4 | (1.4) | (-0.7 to 0.4) | | | 141.6 | (1.8) | (-0.6 to 0.8) | |
| Chlorine (mEq/L) | Placebo | 105.8 | (1.8) | 0.0 | 0.9006 | | 107.0 | (1.5) | 0.1 | 0.7885 |
| | Test | 105.8 | (1.2) | (-0.7 to 0.6) | | | 107.0 | (1.7) | (-0.6 to 0.8) | |
| Potassium (mEq/L) | Placebo | 4.2 | (0.3) | 0.0 | 0.9100 | | 4.3 | (0.4) | -0.1 | 0.4012 |
| | Test | 4.2 | (0.3) | (-0.1 to 0.1) | | | 4.3 | (0.3) | (-0.2 to 0.1) | |
| Calcium (mEq/L) | Placebo | 9.3 | (0.3) | 0.0 | 0.9772 | | 9.1 | (0.3) | 0.0 | 0.4671 |
| | Test | 9.3 | (0.3) | (-0.1 to 0.1) | | | 9.1 | (0.3) | (-0.2 to 0.1) | |
| Total protein (g/dL) | Placebo | 7.3 | (0.3) | 0.0 | 0.6164 | | 7.1 | (0.3) | 0.1 | 0.3197 |
| | Test | 7.4 | (0.3) | (-0.1 to 0.2) | | | 7.2 | (0.4) | (-0.1 to 0.2) | |
| Albumin (g/dL) | Placebo | 4.4 | (0.3) | 0.0 | 0.9421 | | 4.4 | (0.3) | 0.1 | 0.3875 |
| | Test | 4.4 | (0.3) | (-0.1 to 0.1) | | | 4.4 | (0.3) | (-0.1 to 0.2) | |
| Total bilirubin (g/dL) | Placebo | 0.7 | (0.2) | -0.1 | 0.2994 | | 0.7 | (0.3) | 0.0 | 0.8366 |
| | Test | 0.7 | (0.2) | (-0.2 to 0.1) | | | 0.7 | (0.2) | (-0.1 to 0.1) | |
| AST(GOT) (U/L) | Placebo | 22.6 | (5.3) | 0.8 | 0.5748 | | 22.0 | (5.6) | 1.8 | 0.3981 |
| | Test | 23.4 | (8.1) | (-2.1 to 3.8) | | | 23.7 | (12.4) | (-2.4 to 5.9) | |
| ALT(GPT) (U/L) | Placebo | 23.1 | (9.4) | 4.4 | 0.2080 | | 23.1 | (9.9) | 6.0 | 0.2184 |
| | Test | 27.5 | (20.9) | (-2.5 to 11.3) | | | 29.0 | (29.7) | (-3.6 to 15.5) | |
| LDH (U/L) | Placebo | 191.2 | (32.4) | -0.3 | 0.9679 | | 181.5 | (29.2) | 9.0 | 0.1636 |
| | Test | 191.0 | (26.3) | (-12.8 to 12.3) | | | 190.5 | (29.9) | (-3.7 to 21.8) | |
| ALP (U/L) | Placebo | 221.6 | (78.0) | -6.2 | 0.6590 | | 220.4 | (75.6) | -4.3 | 0.7515 |
| | Test | 215.4 | (49.4) | (-34.0 to 21.5) | | | 216.1 | (44.4) | (-31.0 to 22.4) | |
| γ-GTP (U/L) | Placebo | 32.7 | (19.6) | -2.1 | 0.6818 | | 31.2 | (17.6) | -0.7 | 0.8937 |
| | Test | 30.6 | (26.7) | (-12.0 to 7.9) | | | 30.5 | (29.4) | (-11.2 to 9.8) | |
| CK(CPK) (U/L) | Placebo | 100.3 | (57.7) | 13.6 | 0.3332 | | 105.9 | (54.6) | 42.3 | 0.0546 |
| | Test | 113.9 | (71.4) | (-14.1 to 41.3) | | | 148.2 | (130.2) | (-0.9 to 85.6) | |
| White blood cells (×102/μL) | Placebo | 56.3 | (13.9) | -2.7 | 0.3067 | | 53.5 | (13.7) | -1.9 | 0.5247 |
| | Test | 53.6 | (10.7) | (-8.0 to 2.6) | | | 51.7 | (12.9) | (-7.6 to 3.9) | |
| Red blood cells (×105/μL) | Placebo | 47.3 | (3.1) | 1.3 | 0.0840 | | 46.9 | (3.4) | 6.4 | 0.4198 |
| | Test | 48.6 | (3.9) | (-0.2 to 2.8) | | | 47.5 | (3.9) | (-9.3 to 22.2) | |
| Hemoglobim (g/dL) | Placebo | 14.1 | (1.4) | 0.0 | 0.9649 | | 14.1 | (1.5) | -0.2 | 0.5069 |
| | Test | 14.2 | (1.3) | (-0.6 to 0.6) | | | 13.9 | (1.2) | (-0.8 to 0.4) | |
| Hematocrit (%) | Placebo | 43.5 | (3.5) | 0.3 | 0.6722 | | 42.6 | (3.8) | -0.2 | 0.7897 |
| | Test | 43.8 | (3.6) | (-1.2 to 1.9) | | | 42.4 | (3.2) | (-1.7 to 1.3) | |
| Blood platelets (×104/μL) | Placebo | 26.1 | (6.6) | -0.1 | 0.9395 | | 26.7 | (6.1) | -0.1 | 0.9289 |
| | Test | 26.0 | (4.7) | (-2.5 to 2.3) | | | 26.6 | (5.1) | (-2.5 to 2.3) | |
Values were expressed as the mean (SD). HDL: high-density lipoprotein, LDL: low-denstiy lipoprotein, AST: aspartate aminotransferase, ALT: alanine aminotransferase, LDH: lactate dehydorogenase, ALP: alkaline phosphatase, γ-GTP: γ-glutamly transpeptidase, CK creatine kinase.
